# International Pain Outcomes (IPO) – Brazilian Portuguese version: cross-cultural adaptation and psychometric validation

**DOI:** 10.1590/0034-7167-2024-0346

**Published:** 2025-09-01

**Authors:** Marcia Marques dos Santos Felix, Talita Silva Alves Tibola, Maria Beatriz Guimarães Raponi, Ana Cíntia Ribeiro da Silva, Isadora Braga Calegari, Maíla Fidalgo de Faria, Patrícia da Silva Pires, Maria Helena Barbosa

**Affiliations:** I Universidade Federal do Triângulo Mineiro. Uberaba, Minas Gerais, Brazil; II Universidade Federal de Uberlândia. Uberlândia, Minas Gerais, Brazil; III Universidade Federal da Bahia. Vitória da Conquista, Bahia, Brazil

**Keywords:** Pain, Postoperative, Pain Management, Nursing Methodology Research, Validation Study, Psychometrics, Dolor Postoperatorio, Manejo del Dolor, Investigación Metodológica en Enfermería, Estudio de Validación, Psicometría

## Abstract

**Objectives:**

to adapt the International Pain Outcomes questionnaire into Brazilian Portuguese and evaluate its clinimetric properties.

**Methods:**

we conducted a methodological, quantitative, validation, and cross-cultural adaptation study of the instrument. The process included translation, synthesis, expert committee review, back-translation, consensus between the English versions and comparison with the original version, pretest phase (n=30), and metric property validation (convergent validity, n=360; interobserver reliability analysis, n=54; internal consistency, n=360).

**Results:**

the cross-cultural adaptation was successfully performed, and the instrument was considered valid in terms of adequacy and applicability. In the convergent validity analysis, all correlations showed p≤0.001. Cronbach’s alpha was 0.796, Kappa was 1.0 (for dichotomous items), and the Intraclass Correlation Coefficient was >0.999 (for quantitative items).

**Conclusions:**

the instrument was deemed adequate for cultural adaptation, validated for Brazilian Portuguese, and reliable for application in the care of postoperative patients.

## RESUMEN

**Objetivos:**

adaptar al portugués de Brasil y evaluar las propiedades clinimétricas del cuestionario *International Pain Outcomes*.

**Métodos:**

estudio metodológico, cuantitativo, de validación y adaptación transcultural de un instrumento. Se realizaron las siguientes etapas: traducción, síntesis, evaluación por un Comité de Expertos, retrotraducción, consenso de las versiones en inglés y comparación con la versión original, pretest (n=30) y validación psicométrica (validez convergente, n=360; confiabilidad entre observadores, n=54; consistencia interna, n=360).

**Resultados:**

la adaptación transcultural se realizó satisfactoriamente, considerándose el instrumento válido en cuanto a adecuación y aplicabilidad. En la validez convergente, todas las correlaciones presentaron p≤0,001. Se obtuvo un alfa de Cronbach de 0,796, Kappa=1,0 (ítems dicotómicos) y un coeficiente de correlación intraclase > 0,999 (ítems cuantitativos).

**Conclusiones:**

se consideró el instrumento adecuado en términos de adaptación cultural, validado para el portugués de Brasil y confiable para su aplicación en la atención a pacientes en postoperatorio.

## INTRODUCTION

Postoperative pain is a physiological response to tissue damage caused by surgical procedures, leading to an unpleasant physical and psychological experience^(1,2)^. A significant number of patients experience this type of pain despite numerous evidence-based guidelines and protocols, targeted clinical interventions, and standardized assessments^(3,4)^. It is estimated that 240 million patients undergo surgical procedures each year, with 40% to 60% reporting clinically significant pain^(5-8)^.

When inadequately managed, postoperative pain can increase the risk of respiratory and cardiovascular complications, resulting in higher healthcare costs and negatively affecting surgical outcomes and patient satisfaction. It can also lead to postoperative complications that, if unresolved, may increase morbidity and mortality and contribute to disabling chronic pain^(9-11)^. Proper pain management not only provides relief but also facilitates faster patient recovery^(3,12,13)^.

To ensure effective pain management, healthcare professionals must assess pain incidence, its intensity at rest and during daily activities (such as walking, turning in bed, and falling asleep), verify analgesic protocols, monitor adverse effects of medications, evaluate emotional states (such as anxiety, anger, and fear), and assess patient satisfaction, while also incorporating holistic care approaches to pain management^(14,15)^.

To support healthcare professionals in optimizing postoperative pain management in Europe, the PAIN OUT project—an international initiative funded by the European Commission^(16)^ in partnership with the American Pain Society—developed the International Pain Outcomes (IPO) questionnaire. This instrument assesses variability in patient care, identifies best practices in pain management, and helps healthcare providers in decision-making^(17)^.

The IPO questionnaire was designed to evaluate the quality of postoperative pain treatment during the first 24 hours of hospital care and was specifically adapted for the postoperative setting. The most recent English version was revised and validated in 2010^(16)^, and its psychometric properties were assessed in 2013^(18)^.

The cross-cultural adaptation of the IPO to the Brazilian context and the evaluation of its metric properties will enable the availability of an instrument to assist healthcare professionals in assessing pain intensity, severity, and patients’ functional ability after surgery. These applications will contribute to effective postoperative pain management, consequently improving the quality of care, enhancing patient safety, and reducing operational costs for healthcare institutions.

## OBJECTIVES

To adapt the International Pain Outcomes (IPO) questionnaire into Brazilian Portuguese and evaluate its clinimetric properties.

## METHODS

### Ethical aspects

This study followed the recommendations of Resolution 466/2012 of the Brazilian National Health Council. The Research Ethics Committee of the host institution approved it. All participants received verbal and written information about the study objectives and signed the Informed Consent Form (ICF).

### Study design, period, and location

This methodological and quantitative study was conducted in two phases: cross-cultural adaptation and psychometric validation. We carried out it in the Surgical Clinic Unit of a large teaching hospital in the state of Minas Gerais (MG), Brazil. Data collection took place from October to November 2022 (pretest phase) and from February to August 2023 (psychometric validation).

### Population, sample, inclusion, and exclusion criteria

The population involved in the cross-cultural adaptation phase consisted of PhD-level expert judges specializing in the study’s subject and methodology. For the pretest phase and psychometric validation phases, the target population comprised postoperative patients hospitalized in the Surgical Clinic Unit of the host institution.

For the sample size calculation of convergent validity, the required sample was determined based on an expected minimum correlation of 0.2 between the IPO and the Brief Pain Inventory (BPI)^(19)^, with a 95% statistical power and a significance level (alpha) of 0.005. With these parameters, the Power Analysis & Sample Size (PASS) software, version 15, defined the minimum required sample size of 319 participants. However, the final sample included 360 participants.

The sample size calculation for interobserver reliability analysis was based on an expected Intraclass Correlation Coefficient (ICC) of 0.8 between the items of the adapted IPO version, assuming that it would not fall below 0.6, with a 90% statistical power and an alpha of 0.05. Using these parameters in PASS, version 13, the minimum required sample size was 54 patients.

The inclusion criteria for the expert judges were: being Brazilian, having expertise in the study’s subject or methodology, and being clinical nurses directly involved in patient care, all holding a minimum academic degree of PhD and proficiency in English. The judges’ academic credentials were verified through their Lattes Curriculum. Experts who did not return the completed questionnaires within 30 days were considered lost to sample attrition.

The study included patients aged 18 years or older who had undergone elective or emergency surgery, were hospitalized in the Surgical Clinic Unit of the host institution during the data collection period, had been postoperative for at least 24 hours, and were conscious enough to respond to the questionnaire.

Patients unable to engage in dialogue with the researcher were excluded from the study.

### Study protocol

We conducted this study after receiving approval from Dr. Claudia Weinmann, the PAIN OUT project administrator. On April 23, 2018, she granted authorization via email for the instrument to undergo the cross-cultural adaptation process into Brazilian Portuguese.

The IPO questionnaire consists of 18 primary items and 3 secondary items. The primary items assess pain intensity, duration of severe pain, the impact of pain on physical activity, sleep, and emotional state, adverse side effects of treatment, perceived pain relief, level of participation in treatment, and satisfaction with pain management. The secondary items evaluate both the use of non pharmacological approaches for pain relief and the perceived usefulness of information received during treatment. Except for the items measuring duration of severe pain and the extent of pain relief received, which are anchored on a 0% to 100% scale, all primary IPO items are assessed using a numeric rating scale (NRS) ranging from 0 to 10^(17-18)^.

For the cross-cultural adaptation and validation of the instrument, we followed the guidelines proposed by Ferrer et al. (1996)^(20)^ and Beaton et al. (2007)^(21)^.

Two Brazilian translators fluent in English independently translated the instrument. One of them had expertise in the study’s subject matter. The researchers compared both translations, and any discrepancies or ambiguities were clarified with the translators. After analyzing the versions, we synthesized the translations, reaching the first consensus version in Portuguese.

Next, an expert committee of 11 judges evaluated the Portuguese version. This panel included two translators, one postdoctoral researcher in healthcare, five PhD-level nursing professors specializing in the study’s subject and methodology from public universities, and three clinical nurses with PhDs who worked directly in patient care. All had proficiency in English and experience with the study’s topic. Their goal was to assess semantic, idiomatic, cultural, and conceptual equivalences of the items, as well as face and content validity.

We invited the judges via email, which included an invitation letter and the Informed Consent Form (ICF) detailing the purpose and objectives of the study. Those who agreed to participate were required to sign the ICF, scan it, and email it back to us. After receiving their consent, we sent a second email containing both the original English version and the translated Portuguese version for instrument evaluation. The judges then returned the evaluated instrument to us via email.

After all judges returned the evaluated instruments, we assessed content validity through a consensus meeting held via Google Meet with the study authors, the research team, and the expert committee. The judges requested that the original layout be maintained and suggested modifications to the translation. All proposed changes were incorporated, resulting in the Portuguese Consensus Version 1 (PCV1).

We then back-translated PCV1 from Portuguese to English. Two independent translators, native English speakers proficient in Portuguese, conducted the back-translation. They were unfamiliar with the original instrument and had not participated in the initial translation phase. As a result, two new English versions were produced. We compared these versions and synthesized them into a final back-translated version, which was sent to the authors of the original instrument for evaluation.

The document was returned without any modifications, confirming the accuracy of the translation. This step resulted in the Portuguese Pre-Final Version of the IPO, which was then subjected to a pretest. For this phase, we selected a non-probabilistic sample of 30 patients, following the recommendations of the methodological framework adopted. The participants were 18 years or older, in the postoperative period of various surgeries, hospitalized in different surgical and recovery units of the host institution, and had been in the inpatient unit for at least 24 hours postoperatively.

Between October and November 2022, we collected data through self administration of the adapted IPO version and an instrument designed to gather demographic and clinical characterization data. During this phase, participants were encouraged to suggest improvements to the questionnaire whenever they deemed necessary.

Two evaluators assessed convergent validity through independent and simultaneous administration of the IPO and the Brief Pain Inventory (BPI). Data collection took place in the postoperative period (minimum of 24 hours) in a non-probabilistic sample of 360 patients. The empirical evidence for convergent validity was analyzed by examining correlations between conceptually similar items in both instruments (IPO and BPI).

The internal consistency of the items in the adapted version was assessed by calculating the Kuder-Richardson coefficient (KR-20) for dichotomous items and the original Cronbach’s alpha coefficient for quantitative items. Acceptable values ranged between 0.70 and 0.90^(22)^.

To evaluate interobserver reliability, two researchers simultaneously administered the IPO questionnaire to 54 patients, maintaining physical distance from each other and avoiding any communication or exchange of information during data collection. The Kappa coefficient was used for dichotomous items, while the ICC was applied to quantitative items to determine the degree of agreement between the two observers.

## RESULTS

### Cross-cultural adaptation to Brazilian Portuguese

During the translation and synthesis phase, the discrepancies identified between the translations were related to words or terms with similar meanings in Brazilian Portuguese (e.g., surgery/surgical procedure; pain administration/pain management; provide/deliver).

In the expert committee review phase, the members proposed relevant modifications and improvements. After a Google Meet meeting with the study authors, research team, and expert committee members, all suggested changes were discussed, reviewed, and incorporated (Chart 1).

**Chart 1:** Description of modified items after the expert committee review compared to the translated version of the International Pain Outcomes questionnaire

TRANSLATED VERSION		VERSION AFTER EXPERT COMMITTEE REVIEW	
QUESTIONÁRIO SOBRE OS RESULTADOS DO PACIENTE		QUESTIONÁRIO SOBRE OS RESULTADOS DO PACIENTE	
CÓDIGO DO PACIENTE:		CÓDIGO DO PACIENTE	
Caro senhor/senhora Agradecemos se você puder participar da nossa pesquisa sobre como os pacientes se sentem após uma cirurgia. O objetivo da pesquisa é melhorar a administração da dor após cirurgias neste departamento. Sua participação é voluntária, e a informação que você proporcionar vai tornar-se anônima assim que você entregar este questionário. Isso significa que seu nome e outras formas de identificação serão apagados do questionário após a entrega e não serão incluídos em quaisquer registros mantidos por nós. Suas respostas neste questionário não serão compartilhadas com sua equipe médica ou de enfermagem. Sua equipe vai tratá-lo(a) da mesma forma se você escolher participar ou não da pesquisa. Muito obrigado por considerar participar desta pesquisa.		Caro(a) Senhor/Senhora Nós ficaríamos muito gratos pela sua participação em nossa pesquisa sobre como os pacientes se sentem após um procedimento cirúrgico. O objetivo desta pesquisa é melhorar o tratamento da dor pós-operatória nesta unidade. Sua participação é voluntária, e as informações que você fornecer serão armazenadas de forma anônima assim que você entregar este questionário. Isso significa que seu nome e outras formas de identificação serão apagados do questionário após a entrega e não serão incluídos em nenhum dos nossos registros. Suas respostas neste questionário não serão compartilhadas com sua equipe médica ou de enfermagem. A equipe de profissionais que cuida de você vai tratá-lo(a) da mesma forma, independentemente se você escolher participar ou não da pesquisa. Muito obrigado por considerar participar desta pesquisa.	
As questões a seguir são sobre a dor que você sentiu desde a cirurgia.		As questões a seguir são sobre a dor que você sentiu desde a cirurgia.	
P1. Nesta escala, por favor indique a pior dor que você teve desde a cirurgia:		P1. Nesta escala, por favor assinale o número que representa a pior dor que você sentiu desde a cirurgia:	
Sem dor	Pior dor possível	Sem dor	Pior dor possível
P2. Nesta escala, por favor indique a dor menos intensa que você teve desde a cirurgia:		P2. Nesta escala, por favor assinale o número que representa a dor menos intensa que você sentiu desde a cirurgia:	
Sem dor	Pior dor possível	Sem dor	Pior dor possível
P3. Com que frequência você teve dor intensa desde a cirurgia? Por favor, circule sua melhor estimativa da porcentagem de tempo durante o qual você teve dor intensa:		P3. Com que frequência você sentiu dor intensa desde a cirurgia? Por favor, assinale a porcentagem que mais se aproxima do tempo durante o qual você sentiu dor intensa:	
Nunca tive dor intensa	Dor intensa o tempo todo	Nunca senti dor intensa	Dor intensa o tempo todo
P4. Circule o número que melhor descreve o quanto, desde a cirurgia, a dor interferiu ou o(a) impediu de ...		P4. Assinale o número que melhor descreve o quanto, após a cirurgia, a dor interferiu ou o(a) impediu de...	
a. fazer atividades na cama como virar, sentar, mudar de posição:		a. realizar atividades na cama, tais como virar, sentar, mudar de posição:	
Não interferiu	Interferiu totalmente	Não interferiu	Interferiu totalmente
b. respirar profundamente ou tossir:		b. respirar profundamente ou tossir:	
Não interferiu	Interferiu totalmente	Não interferiu	Interferiu totalmente
c. dormir:		c. dormir:	
Não interferiu	Interferiu totalmente	Não interferiu	Interferiu totalmente
d. Você saiu da cama desde a cirurgia?		d. Você conseguiu se levantar da cama após a cirurgia?	
Sim	Não	Sim	Não
Se sim, quanto a dor o(a) atrapalhou ou impediu de fazer atividades fora da cama como caminhar, sentar-se em uma cadeira, ficar em pé à beira da pia:		Se sim, quanto a dor o(a) atrapalhou ou impediu de fazer atividades fora da cama, tais como caminhar, sentar-se em uma cadeira, ir ao banheiro:	
Não interferiu	Interferiu totalmente	Não interferiu	Interferiu totalmente
P5. A dor pode afetar o humor e as emoções.		P5. A dor pode influenciar o nosso humor e as nossas emoções.	
Nesta escala, por favor circule o número que melhor mostra o quanto, desde a cirurgia, a dor fez com que você se sentisse...		Nesta escala, por favor assinale o número que melhor descreve o quanto, após a sua cirurgia, a dor fez com que você se sentisse...	
a. ansioso(a)		a. ansioso(a)	
Nem um pouco	Extremamente	Nem um pouco	Extremamente
b. incapaz		b. desamparado	
Nem um pouco	Extremamente	Nem um pouco	Extremamente
P6. Você teve algum dos seguintes efeitos colaterais desde a cirurgia?		P6. Você percebeu algum dos seguintes efeitos colaterais desde a cirurgia?	
Por favor, circule “0” se não teve; se teve, circule o número que melhor representa a intensidade de cada um:		Por favor, assinale “0” se não sentiu; se sentiu, assinale o número que melhor representa a intensidade de cada um:	
a. Náusea		a. Náusea	
Nenhuma	Severa	Nenhuma	Severa
b. Sonolência		b. Sonolência	
Nenhuma	Severa	Nenhuma	Severa
c. Coceira		c. Coceira	
Nenhuma	Severa	Nenhuma	Severa
d. Tontura		d. Tonturas	
Nenhuma	Severa	Nenhuma	Severa
P7. Desde a cirurgia, quanto alívio para a dor você recebeu?		P7. Desde a cirurgia, quanto alívio para a dor você sentiu?	
Por favor, circule a porcentagem que melhor representa quanto alívio para dor você recebeu de todos os tratamentos para dor juntos (tratamentos medicamentosos ou não):		Por favor, assinale a porcentagem que melhor representa o nível de alívio para dor sentido após todos os tratamentos para dor utilizados (tratamentos medicamentosos e não medicamentosos):	
Nenhum alívio	Alívio completo	Nenhum alívio	Alívio completo
P8. Você gostaria de ter recebido MAIS tratamento para a dor do que recebeu?		P8. Você gostaria de ter recebido MAIS tratamento para a dor do que recebeu?	
Sim	Não	Sim	Não
P9. Você recebeu alguma informação sobre suas opções de tratamento para a dor?		P9. Você recebeu alguma informação sobre as suas opções de tratamento para a dor?	
Sim	Não	Sim	Não
P10. Você foi autorizado a participar das decisões sobre seu tratamento para a dor tanto quanto gostaria?		P10. Você participou das decisões sobre seu tratamento para a dor tanto quanto gostaria?	
De forma alguma	Bastante	Nem um pouco	Bastante
P11. Circule o número que melhor representa o quão satisfeito(a) você está com os resultados do seu tratamento para dor desde a cirurgia:		P11. Assinale o número que melhor representa o quão satisfeito(a) você está com os resultados do seu tratamento para dor desde a cirurgia:	
Extremamente insatisfeito	Extremamente satisfeito	Extremamente insatisfeito	Extremamente satisfeito
P12. Você usou ou recebeu algum método não medicamentoso para alívio da dor?		P12. Você solicitou ou recebeu algum tratamento não medicamentoso para alívio da dor?	
Sim	Não	Sim	Não
Se sim, marque todos que se aplicam:		Se sim, marque todos que se aplicam:	
Bolsa de gelo Meditação Respirar profundamente Calor Acupuntura Prece Conversas com a equipe médica Caminhada Massagem Conversas com amigos ou familiares Relaxamento Imagens ou visualização		Bolsa de gelo Meditação Respiração profunda Calor Acupuntura Orações Conversas com a equipe médica Caminhada Massagem Conversas com amigos ou familiares Relaxamento Imagens ou visualização	
TENS (Neuroestimulação Elétrica Transcutânea)		TENS (Neuroestimulação Elétrica Transcutânea)	
Distrações (como ver TV, ouvir música, ler etc.)		Distrações (como ver TV, ouvir música, ler etc.)	
Outras (por favor, descreva):		Outras (por favor, descreva):	
P13. Você teve alguma condição de dor persistente por três meses ou mais antes de vir ao hospital para a cirurgia?		P13. Antes de vir ao hospital para esta cirurgia você sentiu alguma condição de dor persistente por três meses ou mais?	
Sim	Não	Sim	Não
a. Se sim, quão intensa foi a dor na maior parte do tempo? Por favor, circule o número que indica isso.		a. Se sim, qual a intensidade da dor na maior parte do tempo? Por favor, assinale o número que indica esta intensidade.	
Sem dor	Pior dor possível	Sem dor	Pior dor possível
b. Se teve dor, onde esta dor persistente estava localizada?		b. Se sentiu dor, onde esta dor persistente estava localizada?	
Local da cirurgia Outro local Ambos (local da cirurgia e outro local)		Local da cirurgia Outro local Ambos (local da cirurgia e outro local)	
Obrigado por seu tempo e contribuição		Obrigado por seu tempo e sua participação	
Para ser preenchido pelo assistente de pesquisa Código do assistente de pesquisa: Paciente foi entrevistado: Sim Não Se sim, por favor assinale o(s) motivo(s): Doente/fraco demais Sentia muita dor Necessitava de ajuda Não entendia as escalas Motivos técnicos (paciente estava sem óculos/é cego; não consegue sentar-se; é analfabeto; braço estava engessado etc.)		Para ser preenchido pelo assistente de pesquisa Código do assistente de pesquisa: Paciente foi entrevistado? Sim Não Se sim, por favor assinale o(s) motivo(s): Doente/fraco demais Sentia muita dor Necessitava de ajuda Não entendia as escalas Motivos técnicos (paciente estava sem óculos/é cego; não consegue sentar-se; é analfabeto; braço estava engessado etc.)	

In the back-translation phase, all identified discrepancies were considered synonymous words, and we selected the terms deemed more commonly used in Brazilian Portuguese.

### Pretest

A total of 30 patients participated in the pretest phase. The majority of participants (23; 76.6%) were female, 22 (70.2%) were between 18 and 39 years old, and 8 (29.8%) were between 40 and 60 years old. Additionally, 26 (86%) were married, and 19 (63.2%) had completed high school, while 11 (36.8%) had a higher education degree. Regarding employment status, 21 participants (70%) were employed.

In terms of clinical data, the majority (19; 63.2%) underwent medium- to large-scale surgeries, while 11 (36.3%) had hypertension as a comorbidity. Nineteen (63.2%) received general anesthesia, and no participants experienced operating room complications. All participants received analgesic medication in the operating room and continued with scheduled analgesia for the first 24 hours postoperatively.

**Figure 1:**
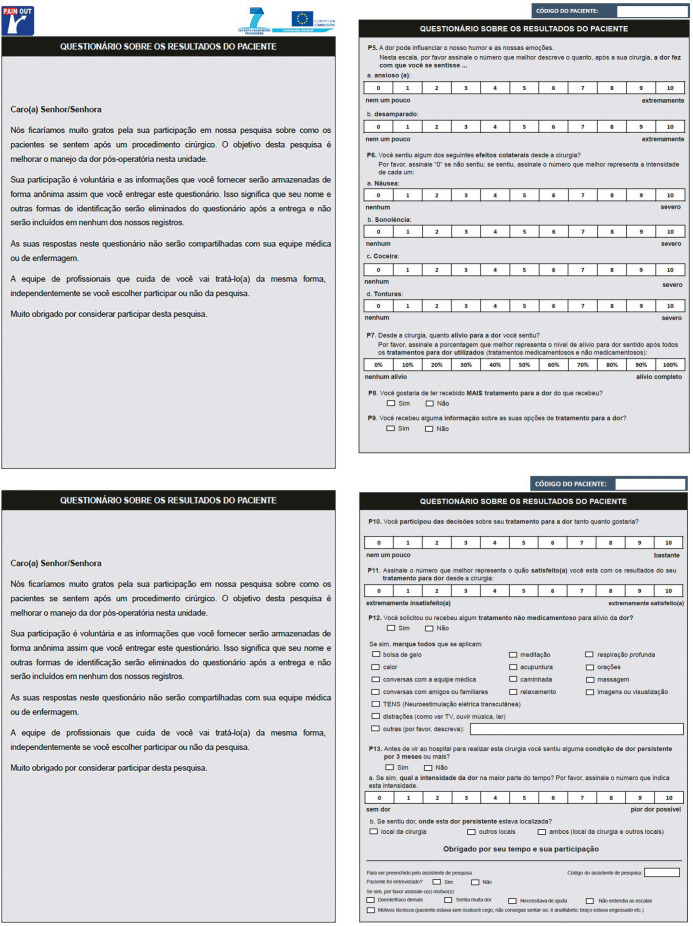
International Pain Outcomes – Brazilian Portuguese version

All pretesting participants reported clearly understanding the instrument’s questions. Based on this result, the instrument was considered validated in terms of adequacy and applicability, leading to the final version of the questionnaire after the pretest, named International Pain Outcomes (IPO) – Brazilian Portuguese version (Figure 1).

### Psychometric validation

Among the 360 participants, 208 (57.8%) were male. The mean age was 55.88 years (±16.37), and the mean surgery duration was 123.44 minutes (±84.39). The results showed that strong and very strong correlations were indicators of convergent validity for conceptually similar items between the instruments analyzed. All correlations between corresponding items in both scales were statistically significant (p ≤ 0.001), demonstrating that the IPO is capable of assessing pain in patients to the same extent as the BPI, an instrument that has already been validated and widely used in clinical practice (Table 1).

**Table 1:** Convergent validity (N = 360) considering the Brief Pain Inventory, Uberaba, Minas Gerais, Brazil, 2023

IPO	BPI	
	R	*p* value
Number representing the worst pain since surgery	Number best describing the worst pain in the last 24 h	
	0.933	≤ 0.001
Number representing the least intense pain since surgery	Number best describing the least intense pain in the last 24 h	
	0.867	≤ 0.001
Number representing how much pain interfered with activities out of bed	Number best describing how much pain interfered with walking ability	
	0.806	≤ 0.001
Number representing how much pain interfered with sleep	Number best describing how much pain interfered with sleep	
	0.786	≤ 0.001
Pain relief level after treatments used	Improvement provided by treatments or medications	
	0.809	≤ 0.001

*IPO – International Pain Outcomes; BPI – Brief Pain Inventory.*

The internal consistency analysis was assessed using the Kuder-Richardson coefficient (KR-20) for dichotomous items and Cronbach’s alpha for quantitative items. For the 0-to-10 scales, Cronbach’s alpha was 0.796, which falls within the acceptable range for instrument reliability.

We also assessed interobserver reliability using Kappa statistics and the ICC between observers for each instrument item. All Kappa results (Table 2) were equal to 1, and the ICC exceeded 0.999, both indicating high interobserver reliability (Table 3).

**Table 2:** Interobserver reliability results (n = 54) using the Kappa coefficient, Uberaba, Minas Gerais, Brazil, 2023

Items	Observer 1				Observer 2				Proportion of agreement (%)	Kappa	*p* value
	Yes		No		Yes		No				
	n	%	N	%	n	%	N	%			
Did you manage to get out of bed?	48	88.9	6	11.11	48	88.9	6	11.11	100	1	< 0.001
Would you have liked to receive more treatment than you did?	10	18.5	44	81.5	10	18.6	44	81.4	100	1	< 0.001
Did you receive information about your pain treatment options?	21	38.9	33	61.1	21	38.9	33	61.1	100	1	< 0.001
Non-pharmacological treatment	9	9.3	45	90.7	9	9.3	45	90.7	100	1	< 0.001
Before this surgery, did you have persistent pain for three months or more?	23	42.6	31	57.4	23	42.6	31	57.4	100	1	< 0.001

**Table 3:** Interobserver reliability results (n = 54) using the Intraclass Correlation Coefficient, Uberaba, Minas Gerais, Brazil, 2023

Itens	Observer 1					Observer 2						*p* value
	Min.	Max.	Mean	Median	SD	Min.	Max.	Mean	Median	SD	ICC	
Worst pain	0	10	5.65	6.5	3.43	0	10	5.65	6.5	3.43	1	< 0.001
Least pain	0	10	2.41	2	2.51	0	10	2.41	2	2.51	1	< 0.001
Frequency of severe pain	0	100	36.85	25	35.97	0	100	36.85	25	35.97	1	< 0.001
Interfered with performing activities in bed	0	10	4.89	5	4.17	0	10	4.89	5	4.17	1	< 0.001
Interfered with breathing or coughing	0	10	3.96	1	4.34	0	10	3.96	1	4.34	1	< 0.001
Interfered with sleeping	0	10	4.13	4.5	4.15	0	10	4.2	4.5	4.20	0.99	< 0.001
Made you feel anxious	0	10	4.33	5	4.05	0	10	4.33	5	4.05	1	< 0.001
Made you feel helpless	0	10	2.28	0	3.61	0	10	2.28	0	3.61	1	< 0.001
Experienced nausea as a side effect	0	10	2.22	0	3.47	0	10	2.22	0	3.47	1	< 0.001
Experienced drowsiness	0	10	4.06	4	4.11	0	10	4.06	4	4.11	1	< 0.001
Experienced itching as a side effect	0	10	2.02	0	3.29	0	10	2.02	0	3.29	1	< 0.001
Experienced dizziness as a side effect	0	10	2.57	0	3.44	0	10	2.57	0	3.44	1	< 0.001
Pain relief from treatments used	0	100	70	90	36.70	0	100	70	90	36.70	1	< 0.001
Participation in pain treatment decisions	0	10	5.85	9.5	4.64	0	10	5.85	9.5	4.64	1	< 0.001
Satisfaction with pain treatment outcomes	0	10	9.33	10	1.64	0	10	9.33	10	1.64	1	< 0.001

## DISCUSSION

Adapting and validating outcome assessment instruments available in the international literature is a viable option as it saves time and resources while enabling comparisons between studies conducted in different countries or among individuals from diverse backgrounds^(21)^.

The cross-cultural adaptation of the IPO for use in Brazil followed a systematic approach, adhering to internationally recommended guidelines for the cultural adaptation of measurement instruments, as described by Ferrer et al.^(20)^ and Beaton et al. (2007)^(21)^. Modifications were made to account for the specific characteristics of the target population during the adaptation process, ensuring that the instrument would be well understood and effectively applied. According to the literature, culturally equivalent translation is essential to ensure that the terms used in the instrument align with the experiences and context of the target population. If these terms are irrelevant to the population’s reality, they should be modified accordingly^(22,23)^.

In this study, modifications to the translated version’s items were guided by expert recommendations and based on principles that reinforce the importance of respecting the autonomy of individuals who have undergone surgical procedures and experienced postoperative pain. The pretest phase reinforced the objective of ensuring clarity and precision in word choice, allowing the translated version to be easily understood by the target population while maintaining the originality of the source instrument^(24,25)^.

Regarding convergent validity, the results indicated a strong to very strong correlation between specific items that shared conceptual similarities across both instruments, demonstrating that the IPO can assess pain in patients to the same extent as the IBD, a validated instrument widely used in clinical practice. In contrast, Erden et al.^(15)^, when combining the IPO questionnaire with the IBD to validate the Turkish version of the IPO, found a moderate positive correlation between similar items in both scales.

In the internal consistency analysis, Cronbach’s alpha was 0.796. This value is similar to that reported in the validation study of the original instrument^(18)^, in which the IPO was applied to 9,727 patients in ten languages across eight European countries and Israel. In that study, the overall alpha was high (0.86), and the psychometric quality of the instrument was deemed satisfactory.

In the validation study of the Turkish version of the IPO, the questionnaire was administered to 250 surgical patients (98 men and 152 women) between January 2015 and January 2016. Consistent with our findings, internal consistency showed a Cronbach’s alpha of 0.88. The authors concluded that the Turkish version of the IPO is a reliable and valid tool for assessing and evaluating the quality of postoperative pain management in the Turkish population^(15)^.

In contrast, the validation study of the Danish version of the IPO evaluated 56 patients with acute abdominal pain, assessing internal consistency through an item-level analysis of the instrument. The results showed a Cronbach’s alpha ≥ 0.7 for the pain, satisfaction, activity, and emotion subscales; 0.69 for the satisfaction subscale in the “hospitalization” domain; ≤ 0.7 for the safety subscale; and ≤ 0.62 for the patient barrier subscale. The study did not report a Cronbach’s alpha for the overall scale^(26)^.

Moreover, in the validation study of the IPO involving 268 Danish and 311 Australian patients, the results demonstrated unsatisfactory internal consistency, with a Cronbach’s alpha of 0.54 for the Danish sample and 0.63 for the Australian sample^(27)^. Supporting these findings, the questionnaire used to validate the Icelandic version was applied to 143 inpatients in a university hospital, yielding a Cronbach’s alpha of 0.42 for the total scale^(28)^.

The level of agreement between observers regarding the instrument’s scores was verified in this study through interobserver analysis, assessed using the Kappa coefficient and the ICC. All Kappa values were equal to 1, and the ICC results exceeded 0.999, indicating high reliability. The approach adopted in this study to evaluate the reliability of the questionnaire following cross cultural adaptation was not used to validate the original instrument. However, it achieved statistically satisfactory results.

Measurements obtained with valid and reliable instruments are essential for identifying effective pain treatment strategies and assessing improvements in pain management quality in clinical practice. The following international studies have reported using the IPO for postoperative pain assessment and management optimization.

To implement quality improvement projects for pain management in different countries, the PAIN OUT group used the IPO to evaluate postoperative pain management in 10,415 patients from 105 surgical wards across 64 hospitals in Mexico, China, and eight European countries. Among these patients, 48.7% reported worst pain scores between 7 and 10, while 24.3% experienced severe pain for more than 50% of the time since surgery, and 34% reported severe functional and emotional interference due to pain. The results indicated that postoperative pain management quality in the assessed countries requires improvement^(29)^.

To report on the development and feasibility of the PAIN OUT project, Zaslansky et al. (2015)^(30)^ employed the IPO questionnaire to evaluate, on the first postoperative day, 6,347 adult patients who had undergone orthopedic or general surgery in eleven medical centers across Europe and Israel. Their findings demonstrated that the PAIN OUT project provides healthcare professionals with standardized and validated instruments for outcome measurement and feedback, supporting their decision-making process to optimize pain management for their patients.

From this perspective, regular pain assessments are recommended after surgery to increase healthcare professionals’ awareness, enabling them to provide patients with specific and effective analgesic approaches^(15)^.

Considering the objectives of the present study, the cross-cultural adaptation process and psychometric validation of the International Pain Outcomes (IPO) questionnaire – Brazilian Portuguese version were conducted appropriately, ensuring its validity.

### Study limitations

One limitation of this study lies in the use of different validation methodologies across distinct cultural contexts. Nevertheless, this study adhered to the validation process established in the original instrument’s development.

### Contributions to the field

This pain assessment instrument can be applied in the Brazilian context, assisting healthcare professionals in evaluating the intensity and severity of postoperative pain, side effects, physical and emotional aspects, functional ability, and self-care of patients after surgery. Its application will support effective postoperative pain management, quality care, and patient safety.

## CONCLUSIONS

The International Pain Outcomes (IPO) questionnaire – Brazilian Portuguese version was considered adequate, culturally equivalent to the original, validated for Brazilian Portuguese, and reliable for application in postoperative patient care. This instrument enables proper and safe pain management, contributing to the prevention of complications during this period.

## Data Availability

The research data are available within the article.
