# Supplementary material for: International Pain Outcomes (IPO) – Brazilian Portuguese version: cross-cultural adaptation and psychometric validation
Source: Rev Bras Enferm. 2025 Sep 1;78(3):e20240346. doi: 10.1590/0034-7167-2024-0346 (PMC12408086; doi:10.1590/0034-7167-2024-0346)
Supplement: Translation

# Translation: *International Pain Outcomes* (IPO) – versão português do Brasil: adaptação transcultural e validação psicométrica

**DOI:** 10.1590/0034-7167-2024-0346pt

**Authors:** Marcia Marques dos Santos Felix, Talita Silva Alves Tibola, Maria Beatriz Guimarães Raponi, Ana Cíntia Ribeiro da Silva, Isadora Braga Calegari, Maíla Fidalgo de Faria, Patrícia da Silva Pires, Maria Helena Barbosa

**Affiliations:** I Universidade Federal do Triângulo Mineiro. Uberaba, Minas Gerais, Brazil; II Universidade Federal de Uberlândia. Uberlândia, Minas Gerais, Brazil; III Universidade Federal da Bahia. Vitória da Conquista, Bahia, Brazil

**Keywords:** Dor Pós-Operatória, Manejo da Dor, Pesquisa Metodológica em Enfermagem, Estudo de Validação, Psicometria

## Abstract

**Objetivos:**

adaptar para a língua portuguesa do Brasil e avaliar as propriedades clinimétricas do questionário *International Pain Outcomes*.

**Métodos:**

pesquisa metodológica, quantitativa, de validação e adaptação transcultural de instrumento. Realizou-se tradução; síntese; avaliação pelo Comitê de Juízes; retrotradução; consenso das versões em inglês e comparação com a versão original; pré-teste (n=30); e validação de propriedades métricas (validade convergente, n=360; análise de confiabilidade interobservadores, n=54; consistência interna, n=360).

**Resultados:**

a adaptação transcultural foi realizada satisfatoriamente, e considerou-se o instrumento validado quanto à adequação e aplicação. Na validade convergente, todas as correlações apresentaram p≤0,001. Obteve se alfa de Cronbach de 0,796, Kappa=1,0 (itens dicotômicos) e coeficiente de correlação intraclasse > 0,999 (itens quantitativos).

**Conclusões:**

considerou-se o instrumento adequado quanto à adaptação cultural, validado para português do Brasil e confiável para aplicação na assistência a pacientes em pós-operatório.

## INTRODUÇÃO

A dor pós-operatória é uma resposta fisiológica ao dano tecidual causado pelo procedimento cirúrgico, levando a uma experiência física e psicológica desagradável^(1,2)^. Um grande número de pacientes vivencia essa dor a despeito das inúmeras diretrizes e protocolos baseados em evidências, intervenções clínicas focadas e avaliações padronizadas^(3,4)^. Estima-se que 240 milhões de pacientes sejam submetidos a cirurgias por ano, dos quais 40% a 60% relatam dor clinicamente significativa^(5-8)^.

Quando mal administrada, a dor pós-operatória pode levar ao risco aumentado de complicações respiratórias e cardiovasculares, acarretando elevação dos custos do cuidado e efeitos negativos nos resultados da cirurgia e na satisfação do paciente. Pode também gerar complicações pós-operatórias, que, se não forem resolvidas, aumentam a morbimortalidade e levam à dor crônica incapacitante^(9-11)^. O manejo adequado da dor não apenas traz alívio, mas também ajuda a acelerar a recuperação do paciente^(3,12,13)^.

Para proporcionar um manejo efetivo da dor, os profissionais de saúde devem avaliar a incidência de dor, sua intensidade em repouso e durante as atividades diárias (como caminhar, virar na cama e adormecer), verificar o protocolo de analgesia, monitorar os efeitos adversos dos medicamentos e o estado emocional (como ansiedade, raiva e medo) e avaliar a satisfação do paciente, estabelecendo também cuidados holísticos no tratamento da dor^(14,15)^.

Para auxiliar os profissionais de saúde a otimizar o gerenciamento da dor pós-operatória na Europa, o PAIN OUT, projeto internacional financiado pela Comissão Europeia^(16)^ em parceria com a Sociedade Americana de Dor, desenvolveu o questionário *International Pain Outcomes* (IPO). Trata-se de um instrumento que mede a variabilidade no atendimento, identifica as melhores práticas de manejo da dor e auxilia os profissionais na tomada de decisões^(17)^.

O questionário IPO foi desenvolvido para medir a qualidade do tratamento da dor pós operatória durante as primeiras 24 horas de atendimento ao paciente no ambiente hospitalar, tendo sido adaptado para o ambiente pós-operatório. A versão em inglês mais recente foi revisada e validada em 2010^(16)^, e a qualidade psicométrica foi avaliada em 2013^(18)^.

A adaptação transcultural para o contexto brasileiro e a avaliação das suas propriedades métricas possibilitarão a disponibilização de um instrumento para ajudar os profissionais de saúde a avaliar a intensidade e gravidade da dor e a capacidade funcional dos pacientes após a cirurgia. Essas aplicações proporcionarão um manejo eficaz da dor pós-operatória e, consequentemente, a melhoria da qualidade da assistência e da segurança do paciente bem como a redução de custos operacionais para as instituições de saúde.

## OBJETIVOS

Adaptar para a língua portuguesa do Brasil e avaliar as propriedades clinimétricas do questionário *International Pain Outcomes* (IPO).

## MÉTODOS

### Aspectos éticos

Este estudo seguiu as recomendações da Resolução 466/2012 do Conselho Nacional de Saúde. Foi submetido e aprovado pelo Comite de Ética em Pesquisa da Instituição sede do estudo. Todos os participantes receberam informações verbais e escritas sobre os objetivos da pesquisa e assinaram o Termo de Consentimento Livre e Esclarecido (TCLE).

### Desenho, período e local do estudo

Este estudo metodológico e quantitativo foi realizado em duas etapas: adaptação transcultural e validação psicométrica. A pesquisa foi realizada no setor de Clínica Cirúrgica de um hospital de ensino de grande porte, no interior do estado de Minas Gerais (MG). As coletas de dados foram realizadas de outubro a novembro de 2022 (pré-teste) e de fevereiro a agosto de 2023 (validação psicométrica).

### População, amostra, critérios de inclusão e exclusão

A população que realizou a adaptação transcultural foi composta de juízes doutores que eram especialistas na temática e no método do estudo. Para o pré-teste e validação psicométrica, a população-alvo foram os pacientes em pós-operatório internados na Unidade de Clínica Cirúrgica da instituição sede do estudo.

Para o dimensionamento amostral da validade convergente, o cálculo do tamanho amostral considerou uma expectativa de correlação mínima de 0,2 entre o IPO e o Inventário Breve de Dor (IBD)^(19)^, um poder estatístico de 95% a ser alcançado e um nível de significância (alfa) de 0,005. Utilizando o aplicativo *Power Analysis & Sample Size* - PASS, versão 15, inserindo-se os parâmetros acima, obteve-se um tamanho amostral mínimo de 319 participantes. No entanto, a amostra final contou com 360 participantes.

O cálculo do tamanho amostral para a análise de confiabilidade interobservador considerou um Coeficiente de Correlação Intraclasse (ICC) esperado de 0,8 entre os itens da versão adaptada do IPO, admitindo-se que não seja inferior a 0,6% para um poder de 90% e alfa igual a 0,05. Inserindo esses valores no aplicativo *Power Analysis and Sample* *Size* (PASS), versão 13, obteve-se um tamanho amostral mínimo de 54 pacientes.

Os critérios de inclusão para os juízes/peritos foram: brasileiros, especialistas na temática ou no método do estudo e enfermeiros clínicos que atuavam diretamente na assistência ao paciente, todos com título mínimo de doutor e domínio da língua inglesa. Os dados relativos à titulação do profissional foram verificados no Currículo Lattes. Os peritos que não devolveram os questionários respondidos no prazo de 30 dias foram considerados como perda de amostragem.

Incluíram-se pacientes com idade igual ou superior a 18 anos, em pós-operatório de cirurgias eletivas ou de emergência, internados na Unidade de Clínica Cirúrgica da instituição sede do estudo durante o período de coleta dos dados, com tempo mínimo de 24 horas de pós-operatório na unidade e que estavam conscientes o suficiente para responder ao questionário.

Excluíram-se os pacientes que não estavam em condições de manter diálogo com a pesquisadora.

### Protocolo do estudo

O estudo foi conduzido após anuência da Dra. Claudia Weinmann, administradora do projeto PAIN OUT. No dia 23/04/2018, a referida autora enviou e-mail dando autorização para que o instrumento fosse submetido ao processo de adaptação cultural para o português do Brasil.

O IPO contém 18 itens primários e 3 secundários. Os itens primários medem intensidade da dor; tempo de dor intensa; impacto da dor na atividade física, no sono e no afeto dos pacientes; efeitos colaterais adversos do tratamento; alívio da dor percebida pelos pacientes; nível de participação permitido no tratamento e satisfação com o tratamento. Itens secundários medem tanto o uso de abordagens não farmacológicas para reduzir a dor quanto a percepção da utilidade das informações recebidas durante o tratamento. Com exceção dos itens que avaliam o tempo de dor intensa e a quantidade de alívio da dor recebida, ancorados entre 0% e 100%, os itens IPO primários são medidos em uma escala de classificação numérica de 0 a 10^(17,18)^.

Para a adaptação transcultural e validação do instrumento, seguiram-se as orientações propostas por Ferrer et al. (1996)^(20)^ e Beaton et al. (2007)^(21)^.

O instrumento foi traduzido por dois tradutores, brasileiros, com proficiência do idioma inglês, e um deles tinha conhecimento sobre a temática avaliada. As traduções foram comparadas pelas pesquisadoras, e as discrepâncias ou ambiguidades existentes foram esclarecidas com os tradutores. Após análise das versões, foi realizada uma síntese das traduções, alcançando-se, assim, o primeiro consenso da versão em português.

Em seguida, a versão em português foi avaliada por um comitê de 11 juízes (2 tradutores, 1 pós-doutora em atenção à saúde, 5 doutoras em enfermagem, docentes pesquisadoras de instituições públicas de ensino especialistas na temática e no método do estudo, e 3 doutores, enfermeiros clínicos, que atuavam diretamente na assistência ao paciente, todos com domínio da língua inglesa e com experiência na temática estudada). O objetivo era verificar as equivalências semântica, idiomática, cultural e conceitual dos itens, bem como a validade de face e conteúdo.

Os juízes foram convidados via e-mail no qual constavam uma carta-convite e o TCLE com a descrição da finalidade e dos objetivos da pesquisa. Caso aceitasse participar, o convidado deveria enviar o TCLE assinado e digitalizado às pesquisadoras via e-mail. Depois de recebido o consentimento dos juízes, as pesquisadoras encaminharam um segundo e-mail com o instrumento em sua versão original e com a versão português, com fins de avaliação do instrumento. Depois, os juízes devolveram o instrumento às pesquisadoras também via e-mail.

Após a devolução de todos os instrumentos pelos juízes, a validade de conteúdo do instrumento foi verificada pelo consenso obtido em reunião (via Google Meet) entre os autores, as pesquisadoras e os profissionais do Comitê de Juízes. Os juízes solicitaram a manutenção do layout original e modificações na tradução do instrumento. Todas as alterações sugeridas por eles foram respeitadas, resultando na Versão Português Consensual 1(VPC1).

A VPC1 foi retrotraduzida do idioma português para o inglês por dois tradutores independentes, ingleses, com domínio da língua portuguesa, que desconheciam o instrumento original e não participaram da primeira etapa do processo de tradução. Após a conclusão dessa etapa, duas novas versões em inglês foram produzidas. Elas foram comparadas pelas pesquisadoras; e, após análise, obteve-se a síntese da retrotradução, que foi enviada para ser avaliada pelos autores do instrumento original.

O documento foi devolvido sem nenhuma modificação. Assim, obteve-se a Versão Português Pré-Final do IPO, que foi submetida ao pré-teste. Para essa fase, foi selecionada uma amostra não probabilística de 30 pacientes, conforme recomendado pelo referencial metodológico utilizado. Eles eram maiores de 18 anos, estavam em pós-operatório de diferentes cirurgias, encontravam-se lotados nas diferentes unidades cirúrgicas e de recuperação da instituição sede do estudo e tinham no mínimo 24 horas de pós-operatório na unidade de internação.

Entre outubro e novembro de 2022, ocorreu a coleta de dados por meio da autoaplicação da versão adaptada do IPO e de um instrumento com dados de identificação e de caracterização sociodemográfica e clínica dos participantes. Nessa etapa, ao responder o instrumento, o participante foi encorajado a sugerir melhoramentos quando considerasse apropriado.

A validade convergente foi avaliada mediante a aplicação independente e simultânea do IPO e IBD por dois avaliadores. A coleta de dados se deu no pós-operatório (no mínimo, 24 horas) em uma amostra não probabilística de 360 pacientes. A análise da evidência empírica de validade convergente considerou as correlações entre os itens específicos que apresentaram a maior similaridade conceitual entre os dois instrumentos (IPO e IBD).

A avaliação da consistência interna dos itens da versão adaptada foi determinada pelo cálculo do coeficiente de Kuder-Richardson (KR-20) para os itens dicotômicos e coeficiente alfa de Cronbach original para itens quantitativos. Consideraram-se aceitáveis valores entre 0,70 e 0,90^(22)^.

Para a avaliar a confiabilidade interobservadores, duas pesquisadoras aplicaram simultaneamente o questionário IPO em 54 pacientes, mantendo distância entre elas e sem se comunicarem ou trocarem informações durante a coleta. Utilizou-se o coeficiente Kappa para itens dicotômicos e o ICC para itens quantitativos a fim de avaliar o grau de concordância entre os dois observadores.

## RESULTADOS

### Adaptação transcultural para o português do Brasil

Na fase de tradução e síntese, as divergências encontradas nas traduções relacionaram-se às palavras ou termos com significados semelhantes na língua portuguesa do Brasil (p.ex., cirurgia/procedimento cirúrgico; administração da dor/manejo da dor; fornecer/proporcionar).

Na fase de avaliação pelo Comitê de Juízes, os integrantes realizaram modificações e melhorias relevantes. Após reunião via Google Meet entre os autores, as pesquisadoras e os profissionais do Comitê, todas as alterações sugeridas foram respeitadas e acatadas (Quadro 1).

**Quadro 1:** Descrição dos itens alterados após avaliação do Comitê de Juízes em comparação com a versão traduzida do questionário *International Pain Outcomes*

VERSÃO TRADUZIDA		VERSÃO APÓS AVALIAÇÃO DO COMITÊ DE JUÍZES	
QUESTIONÁRIO SOBRE OS RESULTADOS DO PACIENTE		QUESTIONÁRIO SOBRE OS RESULTADOS DO PACIENTE	
CÓDIGO DO PACIENTE:		CÓDIGO DO PACIENTE	
Caro senhor/senhora Agradecemos se você puder participar da nossa pesquisa sobre como os pacientes se sentem após uma cirurgia. O objetivo da pesquisa é melhorar a administração da dor após cirurgias neste departamento. Sua participação é voluntária, e a informação que você proporcionar vai tornar-se anônima assim que você entregar este questionário. Isso significa que seu nome e outras formas de identificação serão apagados do questionário após a entrega e não serão incluídos em quaisquer registros mantidos por nós. Suas respostas neste questionário não serão compartilhadas com sua equipe médica ou de enfermagem. Sua equipe vai tratá-lo(a) da mesma forma se você escolher participar ou não da pesquisa. Muito obrigado por considerar participar desta pesquisa.		Caro(a) Senhor/Senhora Nós ficaríamos muito gratos pela sua participação em nossa pesquisa sobre como os pacientes se sentem após um procedimento cirúrgico. O objetivo desta pesquisa é melhorar o tratamento da dor pós-operatória nesta unidade. Sua participação é voluntária, e as informações que você fornecer serão armazenadas de forma anônima assim que você entregar este questionário. Isso significa que seu nome e outras formas de identificação serão apagados do questionário após a entrega e não serão incluídos em nenhum dos nossos registros. Suas respostas neste questionário não serão compartilhadas com sua equipe médica ou de enfermagem. A equipe de profissionais que cuida de você vai tratá-lo(a) da mesma forma, independentemente se você escolher participar ou não da pesquisa. Muito obrigado por considerar participar desta pesquisa.	
As questões a seguir são sobre a dor que você sentiu desde a cirurgia.		As questões a seguir são sobre a dor que você sentiu desde a cirurgia.	
P1. Nesta escala, por favor indique a pior dor que você teve desde a cirurgia:		P1. Nesta escala, por favor assinale o número que representa a pior dor que você sentiu desde a cirurgia:	
Sem dor	Pior dor possível	Sem dor	Pior dor possível
P2. Nesta escala, por favor indique a dor menos intensa que você teve desde a cirurgia:		P2. Nesta escala, por favor assinale o número que representa a dor menos intensa que você sentiu desde a cirurgia:	
Sem dor	Pior dor possível	Sem dor	Pior dor possível
P3. Com que frequência você teve dor intensa desde a cirurgia? Por favor, circule sua melhor estimativa da porcentagem de tempo durante o qual você teve dor intensa:		P3. Com que frequência você sentiu dor intensa desde a cirurgia? Por favor, assinale a porcentagem que mais se aproxima do tempo durante o qual você sentiu dor intensa:	
Nunca tive dor intensa	Dor intensa o tempo todo	Nunca senti dor intensa	Dor intensa o tempo todo
P4. Circule o número que melhor descreve o quanto, desde a cirurgia, a dor interferiu ou o(a) impediu de ...		P4. Assinale o número que melhor descreve o quanto, após a cirurgia, a dor interferiu ou o(a) impediu de...	
a. fazer atividades na cama como virar, sentar, mudar de posição:		a. realizar atividades na cama, tais como virar, sentar, mudar de posição:	
Não interferiu	Interferiu totalmente	Não interferiu	Interferiu totalmente
b. respirar profundamente ou tossir:		b. respirar profundamente ou tossir:	
Não interferiu	Interferiu totalmente	Não interferiu	Interferiu totalmente
c. dormir:		c. dormir:	
Não interferiu	Interferiu totalmente	Não interferiu	Interferiu totalmente
d. Você saiu da cama desde a cirurgia?		d. Você conseguiu se levantar da cama após a cirurgia?	
Sim	Não	Sim	Não
Se sim, quanto a dor o(a) atrapalhou ou impediu de fazer atividades fora da cama como caminhar, sentar-se em uma cadeira, ficar em pé à beira da pia:		Se sim, quanto a dor o(a) atrapalhou ou impediu de fazer atividades fora da cama, tais como caminhar, sentar-se em uma cadeira, ir ao banheiro:	
Não interferiu	Interferiu totalmente	Não interferiu	Interferiu totalmente
P5. A dor pode afetar o humor e as emoções.		P5. A dor pode influenciar o nosso humor e as nossas emoções.	
Nesta escala, por favor circule o número que melhor mostra o quanto, desde a cirurgia, a dor fez com que você se sentisse...		Nesta escala, por favor assinale o número que melhor descreve o quanto, após a sua cirurgia, a dor fez com que você se sentisse...	
a. ansioso(a)		a. ansioso(a)	
Nem um pouco	Extremamente	Nem um pouco	Extremamente
b. incapaz		b. desamparado	
Nem um pouco	Extremamente	Nem um pouco	Extremamente
P6. Você teve algum dos seguintes efeitos colaterais desde a cirurgia?		P6. Você percebeu algum dos seguintes efeitos colaterais desde a cirurgia?	
Por favor, circule “0” se não teve; se teve, circule o número que melhor representa a intensidade de cada um:		Por favor, assinale “0” se não sentiu; se sentiu, assinale o número que melhor representa a intensidade de cada um:	
a. Náusea		a. Náusea	
Nenhuma	Severa	Nenhuma	Severa
b. Sonolência		b. Sonolência	
Nenhuma	Severa	Nenhuma	Severa
c. Coceira		c. Coceira	
Nenhuma	Severa	Nenhuma	Severa
d. Tontura		d. Tonturas	
Nenhuma	Severa	Nenhuma	Severa
P7. Desde a cirurgia, quanto alívio para a dor você recebeu?		P7. Desde a cirurgia, quanto alívio para a dor você sentiu?	
Por favor, circule a porcentagem que melhor representa quanto alívio para dor você recebeu de todos os tratamentos para dor juntos (tratamentos medicamentosos ou não):		Por favor, assinale a porcentagem que melhor representa o nível de alívio para dor sentido após todos os tratamentos para dor utilizados (tratamentos medicamentosos e não medicamentosos):	
Nenhum alívio	Alívio completo	Nenhum alívio	Alívio completo
P8. Você gostaria de ter recebido MAIS tratamento para a dor do que recebeu?		P8. Você gostaria de ter recebido MAIS tratamento para a dor do que recebeu?	
Sim	Não	Sim	Não
P9. Você recebeu alguma informação sobre suas opções de tratamento para a dor?		P9. Você recebeu alguma informação sobre as suas opções de tratamento para a dor?	
Sim	Não	Sim	Não
P10. Você foi autorizado a participar das decisões sobre seu tratamento para a dor tanto quanto gostaria?		P10. Você participou das decisões sobre seu tratamento para a dor tanto quanto gostaria?	
De forma alguma	Bastante	Nem um pouco	Bastante
P11. Circule o número que melhor representa o quão satisfeito(a) você está com os resultados do seu tratamento para dor desde a cirurgia:		P11. Assinale o número que melhor representa o quão satisfeito(a) você está com os resultados do seu tratamento para dor desde a cirurgia:	
Extremamente insatisfeito	Extremamente satisfeito	Extremamente insatisfeito	Extremamente satisfeito
P12. Você usou ou recebeu algum método não medicamentoso para alívio da dor?		P12. Você solicitou ou recebeu algum tratamento não medicamentoso para alívio da dor?	
Sim	Não	Sim	Não
Se sim, marque todos que se aplicam:		Se sim, marque todos que se aplicam:	
Bolsa de gelo Meditação Respirar profundamente Calor Acupuntura Prece Conversas com a equipe médica Caminhada Massagem Conversas com amigos ou familiares Relaxamento Imagens ou visualização		Bolsa de gelo Meditação Respiração profunda Calor Acupuntura Orações Conversas com a equipe médica Caminhada Massagem Conversas com amigos ou familiares Relaxamento Imagens ou visualização	
TENS (Neuroestimulação Elétrica Transcutânea)		TENS (Neuroestimulação Elétrica Transcutânea)	
Distrações (como ver TV, ouvir música, ler etc.)		Distrações (como ver TV, ouvir música, ler etc.)	
Outras (por favor, descreva):		Outras (por favor, descreva):	
P13. Você teve alguma condição de dor persistente por três meses ou mais antes de vir ao hospital para a cirurgia?		P13. Antes de vir ao hospital para esta cirurgia você sentiu alguma condição de dor persistente por três meses ou mais?	
Sim	Não	Sim	Não
a. Se sim, quão intensa foi a dor na maior parte do tempo? Por favor, circule o número que indica isso.		a. Se sim, qual a intensidade da dor na maior parte do tempo? Por favor, assinale o número que indica esta intensidade.	
Sem dor	Pior dor possível	Sem dor	Pior dor possível
b. Se teve dor, onde esta dor persistente estava localizada?		b. Se sentiu dor, onde esta dor persistente estava localizada?	
Local da cirurgia Outro local Ambos (local da cirurgia e outro local)		Local da cirurgia Outro local Ambos (local da cirurgia e outro local)	
Obrigado por seu tempo e contribuição		Obrigado por seu tempo e sua participação	
Para ser preenchido pelo assistente de pesquisa Código do assistente de pesquisa: Paciente foi entrevistado: Sim Não Se sim, por favor assinale o(s) motivo(s): Doente/fraco demais Sentia muita dor Necessitava de ajuda Não entendia as escalas Motivos técnicos (paciente estava sem óculos/é cego; não consegue sentar-se; é analfabeto; braço estava engessado etc.)		Para ser preenchido pelo assistente de pesquisa Código do assistente de pesquisa: Paciente foi entrevistado? Sim Não Se sim, por favor assinale o(s) motivo(s): Doente/fraco demais Sentia muita dor Necessitava de ajuda Não entendia as escalas Motivos técnicos (paciente estava sem óculos/é cego; não consegue sentar-se; é analfabeto; braço estava engessado etc.)	

Na retrotradução, todas as divergências encontradas foram consideradas palavras sinônimas, e as pesquisadoras optaram pelos termos que julgaram mais usuais na língua portuguesa do Brasil.

### Pré-teste

Um total de 30 pacientes participou do pré-teste. A maioria dos participantes (23; 76,6%) eram do sexo feminino, 22 (70,2%) tinham entre 18 e 39 anos, e 8 (29,8%) tinham entre 40 e 60 anos; 26 (86%) eram casados; 19 (63,2%) tinham ensino médio completo, sendo 11 (36,8%) com ensino superior. No tocante à ocupação, 21 (70%) estavam empregados.

Quanto aos dados clínicos, a maioria (19; 63,2 %) dos participantes realizou cirurgia de médio a grande porte, 11 (36,3%) apresentaram hipertensão arterial como comorbidade, 19 (63,2%) receberam anestesia geral, e nenhum participante teve complicações na sala de operações. Todos os participantes receberam medicação analgésica na sala de operações e mantiveram essa medicação de horário nas 24 horas seguintes à cirurgia.

Todos os participantes do pré-teste disseram compreender as questões do instrumento de forma clara. Com esse resultado, considerou-se o instrumento validado quanto à adequação e aplicação, gerando assim a versão final do questionário após o pré-teste, denominada *International Pain Outcomes* (IPO) – versão português do Brasil (Figura 1).

**Figura 1:** *International Pain Outcomes* – versão português do Brasil

### Validação psicométrica

Dos 360 participantes, 208 (57,8%) eram homens. A média de idade foi de 55,88 anos (±16,37). O tempo médio de cirurgia foi de 123,44 minutos (±84,39). Os resultados evidenciaram que a correlação forte e muito forte foram indicadores de validade convergente em itens semelhantes entre os instrumentos analisados. Todas as correlações entre os respectivos itens em ambas as escalas foram estatisticamente significantes (p ≤ 0,001), demonstrando que o IPO é capaz de avaliar a dor nos pacientes na mesma medida que o IBD, instrumento já validado e utilizado na prática clínica (Tabela 1).

**Tabela 1:** Validade convergente (N = 360) considerando o Inventário Breve de Dor, Uberaba, Minas Gerais, Brasil, 2023

IPO	IBD	
	R	Valor de *p*
Número que representa a pior dor desde a cirurgia	Número que melhor descreve a pior dor nas últimas 24 h	
	0,933	≤ 0,001
Número que representa a dor menos intensa desde a cirurgia	Número que melhor descreve a dor mais fraca nas últimas 24 h	
	0,867	≤ 0,001
Número que representa o quanto a dor interferiu nas atividades fora da cama	Número que melhor descreve o quanto a dor interferiu em sua habilidade de caminhar	
	0,806	≤ 0,001
Número que representa o quanto a dor interferiu no sono	Número que melhor descreve o quanto a dor interferiu em seu sono	
	0,786	≤ 0,001
Nível de alívio da dor após tratamentos utilizados	Melhora proporcionada pelos tratamentos ou medicações	
	0,809	≤ 0,001

*IPO – International Pain Outcomes; IBD – Inventário Breve de Dor.*

A análise de consistência interna foi avaliada por meio do coeficiente de Kuder-Richardson (KR-20) para itens dicotômicos e alfa de Cronbach para itens quantitativos. Para as escalas de 0 a 10, obteve-se um alfa de 0,796, dentro dos valores considerados aceitáveis para a confiabilidade do instrumento.

Também se considerou a análise de confiabilidade interobservadores avaliada por meio do Kappa, assim como o ICC entre os observadores para cada item do instrumento. Todos os resultados de Kappa (Tabela 2) foram iguais a 1, e o ICC foi acima de 0,999, ambos os quais indicam boa confiabilidade interobservador (Tabela 3).

**Tabela 2:** Resultados da confiabilidade interobservadores (n = 54) por meio do coeficiente de Kappa, Uberaba, Minas Gerais, Brasil, 2023

Itens	Observador 1				Observador 2				Proporção da concordância (%)	Kappa	Valor de *p*
	Sim		Não		Sim		Não				
	n	%	N	%	n	%	N	%			
Já conseguiu levantar da cama?	48	88,9	6	11,11	48	88,9	6	11,11	100	1	< 0,001
Gostaria de ter recebido mais tratamento do que recebeu?	10	18,5	44	81,5	10	18,6	44	81,4	100	1	< 0,001
Recebeu informações sobre suas opções de tratamento para dor?	21	38,9	33	61,1	21	38,9	33	61,1	100	1	< 0,001
Tratamento não medicamentoso	9	9,3	45	90,7	9	9,3	45	90,7	100	1	< 0,001
Antes dessa cirurgia, apresentou alguma dor persistente por três meses ou mais?	23	42,6	31	57,4	23	42,6	31	57,4	100	1	< 0,001

**Tabela 3:** Resultados da confiabilidade interobservadores (n = 54) por meio do Coeficiente de Correlação Intraclasse, Uberaba, Minas Gerais, Brasil, 2023

Itens	Observador 1					Observador 2						Valor de *p*
	Mín.	Máx.	Média	Mediana	DP	Mín.	Máx.	Média	Mediana	DP	ICC	
Pior dor	0	10	5,65	6,5	3,43	0	10	5,65	6,5	3,43	1	< 0,001
Menor dor	0	10	2,41	2	2,51	0	10	2,41	2	2,51	1	< 0,001
Frequência de dor intensa	0	100	36,85	25	35,97	0	100	36,85	25	35,97	1	< 0,001
Interferiu em realizar atividades na cama	0	10	4,89	5	4,17	0	10	4,89	5	4,17	1	< 0,001
Interferiu em respirar ou tossir	0	10	3,96	1	4,34	0	10	3,96	1	4,34	1	< 0,001
Interferiu em dormir	0	10	4,13	4,5	4,15	0	10	4,2	4,5	4,20	0,99	< 0,001
Fez com que se sentisse ansioso	0	10	4,33	5	4,05	0	10	4,33	5	4,05	1	< 0,001
Fez com que se sentisse desamparado	0	10	2,28	0	3,61	0	10	2,28	0	3,61	1	< 0,001
Teve náusea como efeito colateral	0	10	2,22	0	3,47	0	10	2,22	0	3,47	1	< 0,001
Teve sonolência como efeito colateral	0	10	4,06	4	4,11	0	10	4,06	4	4,11	1	< 0,001
Teve coceira como efeito colateral	0	10	2,02	0	3,29	0	10	2,02	0	3,29	1	< 0,001
Teve tontura como efeito colateral	0	10	2,57	0	3,44	0	10	2,57	0	3,44	1	< 0,001
Alívio da dor com tratamentos utilizados	0	100	70	90	36,70	0	100	70	90	36,70	1	< 0,001
Participação nas decisões do tratamento para dor	0	10	5,85	9,5	4,64	0	10	5,85	9,5	4,64	1	< 0,001
Satisfação com os resultados de tratamento para dor	0	10	9,33	10	1,64	0	10	9,33	10	1,64	1	< 0,001

## DISCUSSÃO

Adaptar e validar os instrumentos para avaliação de resultados disponíveis na literatura internacional torna-se uma opção viável na medida em que permite economizar tempo e recursos bem como comparar os resultados das pesquisas com estudos realizados entre os países ou mesmo entre pessoas de origens distintas^(21)^.

A adaptação transcultural do IPO para uso no Brasil ocorreu de forma sistematizada e seguiu todas as etapas internacionais preconizadas para adaptação cultural de instrumentos de medida, de acordo com o método descrito por Ferrer et al. (1996)^(20)^ e Beaton et al. (2007)^(21)^. Foram realizadas modificações respeitando as particularidades da população-alvo durante a adaptação transcultural, para que o instrumento seja bem compreendido e aplicado de modo eficaz. Isso porque, segundo a literatura, uma tradução culturalmente equivalente é importante para que os termos utilizados no instrumento sejam coerentes com a realidade vivenciada pela população-alvo, dentro de seu contexto cultural. Caso esses termos se encontrem fora do contexto ou da vivência daquela população, eles devem ser modificados^(22,23)^.

Neste estudo, as alterações nos itens da versão traduzida foram guiadas pelas sugestões dos juízes e pautadas nos princípios que reafirmam a importância da autonomia das pessoas que passaram por procedimentos cirúrgicos e vivenciaram a dor pós-operatória. A aplicação do pré-teste sustentou o objetivo de assegurar a compreensão e a escolha precisa das palavras componentes da versão traduzida, garantindo que sejam entendidas com clareza pela população-alvo e preservando a originalidade do instrumento de origem^(24,25)^.

Quanto à validade convergente, os resultados indicaram uma correlação de forte a muito forte entre os itens específicos que apresentaram maior similaridade conceitual entre os dois instrumentos, demonstrando que o IPO é capaz de avaliar a dor nos pacientes na mesma medida que o IBD, instrumento já validado e utilizado na prática avaliada. Já Erden et al.^(15)^, ao conjugarem o questionário IPO com o IBD na validação da versão turca do IPO, encontraram correlação positiva moderada entre os itens similares das duas escalas.

Na análise da consistência interna, o alfa de Cronbach foi de 0,796. O valor se assemelha ao encontrado em estudo de validação do instrumento original^(18)^, em que o questionário IPO foi aplicado a 9.727 pacientes em dez idiomas, abarcando oito países europeus e Israel. O alfa alcançou o nível geral alto (0,86), e a qualidade psicométrica do instrumento foi considerada satisfatória.

No estudo de validação da versão turca do IPO, o questionário foi aplicado a 250 pacientes cirúrgicos (98 homens, 152 mulheres) entre janeiro de 2015 e janeiro de 2016. Alinhado com os nossos resultados, a consistência interna apresentou alfa de Cronbach de 0,88. Os autores concluíram que a versão turca do IPO é confiável e válida para medir e avaliar a qualidade do tratamento da dor pós-operatória na população turca^(15)^.

Em contrapartida, o estudo de validação da versão dinamarquesa do IPO avaliou 56 pacientes com dor abdominal aguda, realizando os testes de consistência interna por meio de avaliação dos itens do instrumento. Os resultados mostraram alfa de Cronbach ≥ 0,7 para as subescalas de dor, satisfação, atividade e emoção; 0,69 para a subescala de satisfação na “internação hospitalar”; ≤ 0,7 para a subescala de segurança; e ≤ 0,62 para a subescala de barreira do paciente. O estudo não apresentou alfa de Cronbach para a escala total^(26)^.

Ainda, no estudo de validação do IPO para 268 pacientes dinamarqueses e 311 pacientes australianos, os resultados demonstraram que a consistência interna foi insatisfatória, com alfa igual a 0,54 para os dinamarqueses e 0,63 para os australianos^(27)^. Corroborando esses resultados, o questionário usado na validação da versão islandesa foi aplicado em 143 pacientes internados nas enfermarias em um hospital universitário, e os resultados apresentaram alfa de Cronbach igual a 0,42 para a escala total^(28)^.

O grau de concordância entre os observadores quanto aos escores do instrumento foi verificado neste estudo por meio da análise interobservadores, avaliada pelo Kappa e pelo ICC. Todos os resultados de Kappa foram iguais a 1, e o ICC apresentou resultados acima de 0,999, indicando alta confiabilidade. A abordagem adotada neste estudo para avaliar a confiabilidade de um questionário após a adaptação transcultural não foi usada na validação do instrumento original, entretanto atingiu resultados estatísticos satisfatórios.

Medições com instrumentos válidos e confiáveis são essenciais para explorar estratégias eficazes de tratamento da dor e avaliar a melhoria da qualidade no manejo da dor na prática clínica. A aplicação do IPO para registrar e avaliar a dor pós-operatória bem como otimizar sua gestão é relatada nos estudos internacionais descritos a seguir.

Visando realizar projetos de melhoria da qualidade no manejo da dor em cada país, o grupo PAIN OUT usou o IPO para avaliar o manejo da dor pós-operatória em 10.415 pacientes de 105 enfermarias cirúrgicas, em 64 hospitais do México, China e oito países europeus. De todos os pacientes, 48,7% relataram pior dor com escore entre 7 e 10, enquanto 24,3% relataram sentir dor intensa por mais de 50% do tempo desde a cirurgia e 34% relataram classificações severas de interferência funcional e emocional relacionadas à dor. Os resultados demonstraram que a qualidade no manejo da dor pós-operatória nos países avaliados necessita melhorias^(29)^.

Para relatar o desenvolvimento e a viabilidade do projeto PAIN OUT, Zaslansky et al. (2015)^(30)^ empregaram o questionário IPO para avaliar, no primeiro dia de pós-operatório, 6.347 pacientes adultos submetidos a cirurgia ortopédica ou geral em onze centros médicos na Europa e em Israel. Os resultados demonstraram que o projeto PAIN OUT oferece aos profissionais da saúde instrumentos padronizados e validados para medição e feedback da qualidade dos resultados, apoiando-os no processo de tomada de decisão para otimizar o manejo da dor de seus pacientes.

Desse panorama, observa-se que avaliações regulares da dor são recomendadas após a cirurgia para que haja um aumento de conscientização dos profissionais de saúde, permitindo que eles forneçam abordagens analgésicas específicas e eficazes ao paciente^(15)^.

Considerando os objetivos do presente estudo, o processo de adaptação transcultural e a validação psicométrica do questionário *International Pain Outcomes* (IPO): versão português do Brasil foram realizados de forma adequada e a validade está assegurada.

### Limitações do estudo

Considera-se como limitação deste estudo as metodologias de validação distintas usadas nas diferentes culturas, entretanto, ressalta-se que este estudo seguiu a validação proposta pelo estudo de validação do instrumento original.

### Contribuições para a Área

Este instrumento de avaliação da dor poderá ser aplicado no contexto brasileiro, auxiliando os profissionais de saúde a avaliar a intensidade e gravidade da dor pós-operatória, efeitos colaterais, aspectos físicos e emocionais, capacidade funcional e autocuidado dos pacientes após a cirurgia. Sua aplicação favorecerá manejo eficaz da dor pós-operatória, assistência de qualidade e segurança ao paciente.

## CONCLUSÕES

O questionário *International Pain Outcomes* (IPO) – versão português do Brasil foi considerado adequado, equivalente ao original quanto à sua adaptação cultural, validado para o português do Brasil e confiável para a aplicação na assistência a pacientes em pós-operatório. Esse instrumento possibilita o manejo adequado e seguro da dor, contribuindo na prevenção de complicações nesse período.

## Data Availability

Os dados da pesquisa estão disponíveis no corpo do artigo.
